# *De Novo* Transcriptome Analysis Shows That SAV-3 Infection Upregulates Pattern Recognition Receptors of the Endosomal Toll-Like and RIG-I-Like Receptor Signaling Pathways in Macrophage/Dendritic Like TO-Cells

**DOI:** 10.3390/v8040114

**Published:** 2016-04-21

**Authors:** Cheng Xu, Øystein Evensen, Hetron Munang’andu Mweemba

**Affiliations:** Section of Aquatic Medicine and Nutrition, Department of Basic Sciences and Aquatic Medicine, Faculty of Veterinary Medicine and Biosciences, Norwegian University of Life Sciences, Ullevålsveien 72, P.O. Box 8146 Dep NO-0033 Oslo, Norway; cheng.xu@nmbu.no (C.X.); oystein.evensen@nmbu.no (Ø.E.)

**Keywords:** dendritic cells, macrophages, Pattern recognition receptor (PRR), RIG-I-like receptor (RLR), Salmonid alphavirus subtype 3 (SAV-3), RNA sequencing (RNA-Seq), Toll-like receptor (TLR), TO-cells

## Abstract

A fundamental step in cellular defense mechanisms is the recognition of “danger signals” made of conserved pathogen associated molecular patterns (PAMPs) expressed by invading pathogens, by host cell germ line coded pattern recognition receptors (PRRs). In this study, we used RNA-seq and the Kyoto encyclopedia of genes and genomes (KEGG) to identify PRRs together with the network pathway of differentially expressed genes (DEGs) that recognize salmonid alphavirus subtype 3 (SAV-3) infection in macrophage/dendritic like TO-cells derived from Atlantic salmon (*Salmo salar* L) headkidney leukocytes. Our findings show that recognition of SAV-3 in TO-cells was restricted to endosomal Toll-like receptors (TLRs) 3 and 8 together with *RIG-I*-like receptors (RLRs) and not the nucleotide-binding oligomerization domain-like receptors NOD-like receptor (*NLRs*) genes. Among the RLRs, upregulated genes included the retinoic acid inducible gene I (RIG-I), melanoma differentiation association 5 (MDA5) and laboratory of genetics and physiology 2 (LGP2). The study points to possible involvement of the tripartite motif containing 25 (TRIM25) and mitochondrial antiviral signaling protein (MAVS) in modulating RIG-I signaling being the first report that links these genes to the RLR pathway in SAV-3 infection in TO-cells. Downstream signaling suggests that both the TLR and RLR pathways use interferon (IFN) regulatory factors (IRFs) 3 and 7 to produce IFN-a2. The validity of RNA-seq data generated in this study was confirmed by quantitative real time qRT-PCR showing that genes up- or downregulated by RNA-seq were also up- or downregulated by RT-PCR. Overall, this study shows that *de novo* transcriptome assembly identify key receptors of the TLR and RLR sensors engaged in host pathogen interaction at cellular level. We envisage that data presented here can open a road map for future intervention strategies in SAV infection of salmon.

## 1. Introduction

A crucial step in cellular defense mechanisms against viral infection is recognition of danger signals that initiate signaling pathways aimed at protecting host cells against pathogen invasion [1]. Apart from protecting host cells, recognition of microbial danger signals is a crucial step for targeted delivery of vaccine antigens into antigen presenting cells (APCs) as recently pointed out by Munang’andu and Evensen [2]. The major players in recognition of microbial invasion are pattern recognition receptors (PRRs) made of germ line coded receptors that recognize conserved microbial features called “pathogen associated molecular patterns” (PAMPs) [3]. In addition, PRRs also recognize endogenous host structures released after tissue damage called “damage associated molecular patterns” (DAMPs) [4]. The numbers of germ line coded PRRs is limited and as such PAMPs represent unique structures that are characteristic of several groups of pathogens.

Currently, there are different PRR families identified in vertebrates that serve as immune sensors of PAMPs and these include the Toll-like receptors (TLRs), retinoic acid-inducible gene I (RIG-I)-like receptors (RLRs), nucleotide oligomerization domain (NOD)-like receptors (NLRs), as well as the melanoma 2 (AIM2) like receptors (ALRs) and the cytoplasmic double stranded DNA sensors (CDSs) [5,6,7,8]. Members of the TLR family detect PAMPs from protozoa, bacteria, fungi and viruses and they can broadly be classified into TLRs found on cell surfaces and those found in endosomal compartments [1,9,10]. The NLRs with known functions mainly recognize bacteria while RLR are antiviral [11]. Thus far, 17 members of the TLRs have been identified in different fish species [12,13] while a genomic overview of NLRs found in fish was recently published by Laing *et al.* [14]. In addition, MDA5 and RIG-I receptors were recently cloned and characterized in salmonids [15]. However, there is little information regarding the signaling pathways induced by these PRRs in different fish species although several genes involved in the downstream signaling of PRR pathways have been cloned and characterized in different fish species [16,17]. One of the major drawbacks to elucidating the signaling pathways induced by different PRRs in fish is the general absence of tools such as knockout models that can be used to elucidate the functional roles of different genes expressed at different stages of the signaling pathways. While the search for signaling pathway analytical tools continues in fish, the emergence of RNA-seq has opened a new dimension in functional genomics in which a vast array of genes expressed in response to host–pathogen interaction can be sequenced at the same time thereby allowing for a global understanding of cellular responses induced by microbial invasion at transcript level [18]. As such, genome wide transcriptome data analysis can be used to identify networks of genes expressed in response to microbial invasions at the same time.

Hence, in the present study, we used a *de novo* assembly to generate a transcriptome of differentially expressed genes (DEGs) generated in response to salmonid alphavirus subtype 3 (SAV-3) infection in macrophages/dendritic like TO-cells derived from Atlantic salmon (*Salmo salar* L) headkidney leukocytes [19,20]. By using the Kyoto encyclopedia of genes and genomes (KEGG) pathway analysis, we wanted to find out the repertoire of genes linked to PRR pathways induced by SAV-3 infection in TO-cells. SAV-3 is the etiological agent for pancreas disease (PD) known to cause high economic losses in salmonids [21,22]. It is a member of the genus alphavirus in the family Togaviridae [23]. It contains a positive sense single stranded RNA (+ssRNA) genome with capped 5′ end and polyadenylated 3′ end that serves directly as messenger RNA (mRNA) for the translation of viral non-structural proteins upon entry and form the dsRNA intermediate during replication in infected cells [24,25]. As pointed out elsewhere [26,27], ssRNA is sensed by RIG-I and TLR-7/8, while dsRNA is sensed by TLR3, RIG-I and MDA5 [27,28,29,30]. The SAV-3 genome is subdivided into two open reading frames (ORFs). The first ORF encodes four non-structural proteins (nsPs) designated as nsP1-4 responsible for the transcription and replication of the viral RNA while the second ORF encode the structural proteins PE2-6K-E1 [31]. Based on the transcriptome analysis presented here, we demonstrate that the repertoire of *PRR* genes expressed in response to SAV-3 infection in TO-cells is comparable to the profile of genes linked to PRR signaling pathways induced by other alphavirus infections in mammalian cells. Further, we also show that pathway based analysis provides a contextual understanding of the biological relevance of DEGs expressed in a transcriptome. We envision that data presented here shall broaden our understanding of the cellular mechanisms used by fish cells to combat microbial invasion.

## 2. Materials and Methods

### 2.1. Cell Culture and Virus Infection

TO-cells derived from Atlantic salmon headkidney leukocytes characterized to possess macrophage/dendritic cell like properties [19,20], were propagated at 20 °C in HMEM (Eagle’s minimal essential medium (MEM) with Hanks’ balanced salt solution (BSS)) supplemented with l-glutamine, MEM nonessential amino acids, gentamicin sulphate, and 10% fetal bovine serum (FBS). When the cells were 80% confluent, one batch was inoculated with SAV-3 (Genebank accession JQ799139) [32] at multiplicity of infection (MOI) 1 while another batch was only exposed to the HMEM growth media. Thereafter, both the SAV-3 infected and non-infected TO-cells were incubated at 15 °C in HMEM maintenance media supplemented with 2% FBS. Cells from both the infected and non-infected groups were harvested after 48 h. Both the SAV-3 infected and non-infected cells were propagated in triplicates.

### 2.2. Total RNA Isolation

Extraction of total RNA from SAV-3 infected and non-infected TO-cells was carried out using the RNAeasy mini kit with on-column DNase treatment according to the manufacturers’ instructions (Qiagen, Hilden, Germany). The quality and concentration of RNA was analyzed using the ND1000 nanodrop (Nanodrop Technologies, Wilmington, NC, USA) and Agilent 2100 Bioanalyzer (Agilent Technologies, Santa Clara, CA, USA).

### 2.3. Library Construction, Sequencing and Data Analysis for RNA-Seq

Library construction was carried out by pooling together triplicate samples obtained from total RNA extraction of SAV-3 infected and non-infected cells for RNA-Seq. Treatment of total RNA with DNase I to degrade any possible DNA contamination, enrichment using oligo(dT) magnetic beads, fragmentation into approximately 200 bp fragments, synthesis of first strand cDNA using random hexamer-primers followed by synthesis of the second strand together with end reparation coupled with 3′-end single nucleotide A (adenine) addition, ligation of sequence adaptors to the fragments and fragment enrichment by PCR amplification were also carried out as previously described in our studies [17]. Thereafter, quality check (QC step) was carried out using the Agilent 2100 Bioanaylzer and ABI StepOnePlus Real-Time PCR System (Bio-Rad) to qualify and quantify the sample library. Subsequently, library products were used for RNA-sequencing using Illumina HiSeqTM 2000, BGI-Hong Kong and clean reads were obtained after removal of adaptor sequences together with reads having >10% of unknown bases and reads with low quality bases (base with quality value ≤5) >50% in a read.

### 2.4. De Novo Assembly, Functional Annotation and Gene Ontology Classification

Once a library of clean reads was prepared, clean reads were then used for *de novo* transcriptome assembly using the Trinity software [33]. Thereafter, the assembled unigenes were annotated into different functional classifications after searching in different protein databases using the BlastX (version 2.2.23) alignment. The four public protein databases used include: (i) NCBI non-redundat (NR); (ii) Swiss-Prot; (iii) Kyoto Encyclopedia of Genes and Genomes (KEGG); and (iv) Cluster of Orthologous Groups (COG) at *e*-value < 0.00001. The direction of the identified unigenes was determined using the best alignments obtained from the four databases. In the case of conflicting results between different databases, the priority order: (i) NR; (ii) Swissprot; (iii) KEGG; and (iv) COG was used. BlastX data was used to extract the coding regions (CDS) from unigene sequences and translate them into peptide sequences. Unigenes not identified by BlastX were analyzed using ESTScan to predict their CDS and to decide their sequence direction while unigenes with NR annotation were further analyzed with Blast2go [34] to obtain their gene ontology (GO) annotations. The identified unigenes were classified according to GO functions using the Web Gene Ontology (WEGO) annotation software.

### 2.5. Identification of Differentially Expressed Genes

Mapped read counts for each gene generated from the functional annotation above were normalized for RNA length and total read counts in each lane using the reads per kilobase per million method (RPKM). As such, the RPKM method allowed for direct comparison of the number of transcripts between the SAV-3 infected and non-infected groups, which created the basis for identifying the differentially expressed genes (DEGs). We set the cutoff limit at 95% confidence interval for all RPKM values for each gene and used a rigorous algorithm to generate DEGs by comparing RPKM mapped reads from SAV-3 infected *versus* non-infected TO-cells. Only DEGs with a threshold of false discovery rate (FDR) <0.001 and an absolute value log_2_ ratio >1 were considered differentially expressed. Thereafter, all identified DEGs were mapped to GO annotations using the Blast2GO software [34] and were later assigned KEGG ortholog (KOs) identifiers for pathway analysis using the KEGG pathway analytical software using the zebrafish model.

### 2.6. Data Access

The RNA-sequencing data generated in this study have been deposited in the National Center for Biotechnology Information (NCBI) Gene Expression Omnibus (GEO) database accession number GSE64095 (www.ncbi.nih.gov/geo Accession number GSE64095) [35].

### 2.7. Validation of RNA-Seq Data and Virus Quantification

In order to confirm the validity of our RNA-seq data, 13 randomly selected DEGs shown to be up- or downregulated by RNA-seq were used for quantitative real-time PCR (qRT-PCR) analysis using the QuantiFast SYBR Green RT-PCR Kit (Qiagen) and the LightCycler 480 system (Roche). For each gene, the quantity of template, master mix final volume, reverse transcriptase, PCR initiation activation and cycles used per reaction were carried out as previously described [17]. Primer sequences used for RT-PCR are shown in Table 1. The specificity of each PCR product from each primer pair was confirmed by melting curve analysis and agarose gel analysis while the 2−^∆∆^^Ct^ method was used to quantify the fold increase in gene expression levels relative to the control group. All quantifications were normalized using the β-actin endogenous gene, which has been shown to be a stable normalizer of different viral infections in Atlantic salmon in our studies [32,36,37]. For virus quantification, qRT-PCR was used to determine the quantity of virus in the SAV-3 infected and non-treated cells using the E2 SP expressed during virus replication using primer sequences used for E2 quantification are shown in Table 1 as previously described by Xu *et al.* [24].

## 3. Results

### 3.1. Gene Ontology Classification and KEGG Pathway Analysis

After filtration, a total of 20,115 unigenes were identified and assigned KOs identifiers. Analysis of DEGs using KOs resulted into 9315 genes being assigned to 252 pathways, which included the PRR pathways shown in Table 2. The significance of each pathway was set at *p*-value <0.05 while the cutoff for enrichment was set at Qvalue <0.50. The TLR pathway had the highest number of DEGs (112) followed by the NLR (89) and RLR pathways (79). Although the TLR pathway had a marginal significance (*p* = 0.058), it was more significant than the NLR pathway (*p* = 0.91), while the RLR pathway showed the highest significance (*p* = 0.024) of all the PRRs expressed in response to SAV-3 infection in TO-cells. As a result, the RLR pathway had the highest enrichment (Qvalue = 3.0117 × 10^−1^) followed by the TLR pathways (Qvalue = 4.8652 × 10^−1^) suggesting that both pathways played a pivotal role in the recognition of SAV-3 infection in TO-cells. On the contrary, the NLR pathway was not enriched (Qvalue = 1.0 × 10^0^) indicating that NLRs had no capacity to recognize the invasion of SAV3 in TO-cells (Table 2).

Hence, in the next studies we focused on the RLRs and TLRs that were significantly enriched to identify the exact sensors within these PRRs that were able to recognize SAV3 infection in TO-cells. To identify the genes involved in downstream signaling after ligand binding of TLRs and RLRs to the SAV-3 PAMPs, we further analyzed the repertoire of DEGs expressed in each pathway as shown below.

### 3.2. Toll-Like Receptor Signaling Pathways

**Figure 1:**
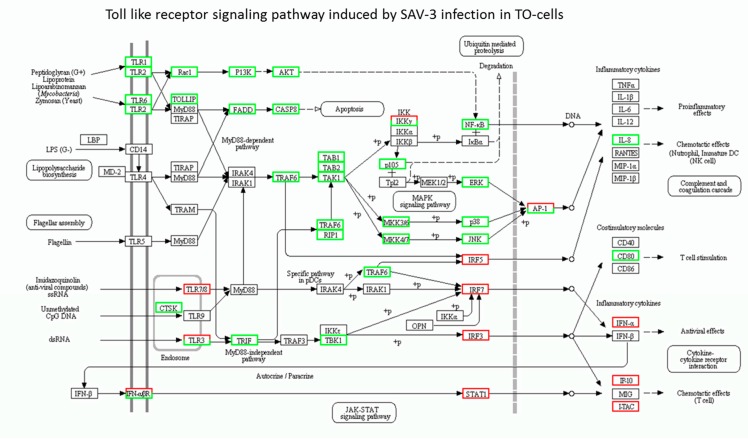
The KEGG pathway analysis for the Toll like receptor (TLR) signaling pathway differentially expressed genes (DEGs) expressed in response to SAV-3 infection in TO-cells. Red squares show unpregulated genes while green squares represent downregulated genes. Square having both red and green represent a mixed expression of upregulated (red) and downregulated (green) unigenes for the gene represented. Black squares show that the genes represented were not expressed in TO-cells.

Table 3A shows the profile of genes upregulated in the TLR signaling pathway induced by SAV3 infection in TO-cells. Only the endosomal TLRs 3 and 8 were upregulated in which the fold increase for TLR8 was approximately threefold higher than TLR3. Among the IFN regulatory factors (IRFs), upregulation of IRF7 was at similar level as IRF3 (Table 3A). Other genes upregulated include IFN α/β receptor 1 (IFNAR1), IFN-a2, and the chemokines IFNγ induced protein 10 (IP-10) and IFN-inducible T-cell α chemoattractant (I-TAC). Figure 1 shows the KEGG network pathways in which only endosomal TLRs 3 and 8 were upregulated, as shown in Table 3A. Downstream signaling from the endosomal TLRs 3 and 8 show upregulation of IRF3 and IFR7 linked to upregulation of IFN-a2. In addition, Figure 1 also shows upregulation of the IFN-α/β receptor linked to upregulation of IP-10 and I-TAC.

**Figure 2:**
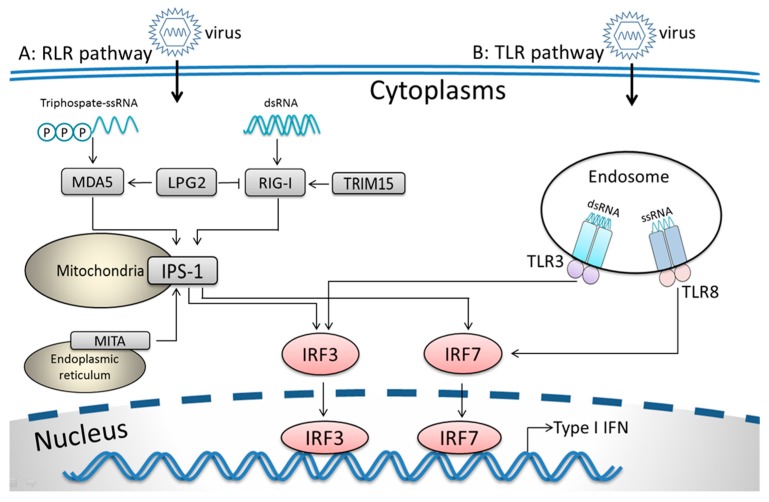
A summary of the Toll like receptors (TLR) and RIG-I like receptor (RLR) pathway genes based on upregulated genes shown in Table 3A and Table 4A excluding the downregulated genes shown in Table 3B and Table 4B. Pathway A shows the RLR signaling pathway, while Pathway B shows the TLR signaling pathway.

To summarize the TLR pathways induced by SAV-3 infection in TO-cells, Figure 2 shows the TLR signaling pathway based on upregulated genes (Table 3A) excluding the downregulated genes (Table 3B).

Table 3B shows downregulated TLR pathway genes in TO-cells infected by SAV-3 in which the extracellular TLRs 1 and 2 were downregulated together with their downstream signaling genes belonging to the P13K-AKT and FADD-CASP8 pathways. In addition, Figure 1 shows that TLRs 4 and 5 were not expressed together with their downstream signaling genes like the translocating chain-associated membrane protein (TRAM), adaptor protein (TIRAP) and myeloid differentiation primary response protein (MyD88) in TO-cells infected by SAV-3. Put together, these data show that none of the extracellular TLRs were upregulated in response to SAV-3 infection in TO-cells.

### 3.3. RIG-I-Like Receptor Signaling Pathway

**Figure 3:**
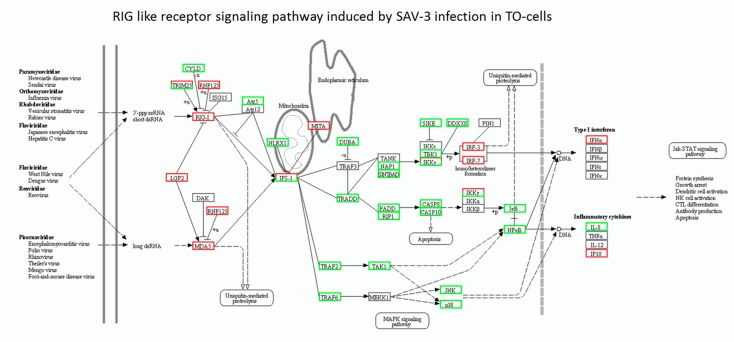
The KEGG pathway analysis of the RIG-I-like receptor DEGs expressed in response to SAV-3 infection TO-cells. Red squares show upregulated genes while green squares represent downregulated genes. Square having both red and green represent a mixed expression of upregulated (red) and downregulated (green) unigenes for the gene represented. Black squares show that the genes represented were not expressed in TO-cells.

Table 4A shows RLR pathway genes that were upregulated in response to SAV-3 infection in TO-cells of which LPG2 had the highest expression followed by RIG-1 and MDA5, respectively. Downstream signaling showed upregulation of the endoplasmic reticulum mediator of IRF3 activation (MITA) and mitochondria IFNβ promoter stimulator I (IPS-I), which is also known as the mitochondrial antiviral-signaling protein (MAVS). Among the genes that regulate the expression of RIG-I, the tripartite motif-containing protein 25 (TRIM25) was upregulated when Ubiquitin carboxyl-terminal hydrolase CYLD was downregulated. Other upregulated genes included IRF3, IRF7, IP-10 and IFN-a2. Figure 3 shows genes differentially expressed for the RLR pathway induced by SAV-3 infection in TO-cells in which RIG-I and MDA5 were upregulated. In addition, Figure 3 shows that TRIM25 was linked to upregulation of RIG-I when CYLD was downregulated. Further, Figure 3 also shows that downstream signaling of RIG-I and MDA5 converge on the interferon-beta promoter stimulator 1 (IPS-I), which is linked to MITA found in the endoplasmic reticulum while downstream signaling from IPS-1 via IRF3 and IRF7 culminated in upregulation of IFN-a2. To summarize the RLR signaling pathway induced by SAV-3 infection in TO-cells, Figure 2 shows a pathway based on upregulated RLR genes (Table 4A) excluding the downregulated genes. Table 4B shows the RLR genes that were downregulated in TO-cells during SAV-3 infection. Consistent with the TLR pathways (Table 3B and Figure 1), genes involving the FADD-CASP8 signaling pathways were downregulated together with genes that signal via the TNF-receptor associated factor 2 (TRAF2) and 6 (TRAF6) pathways.

### 3.4. Quantitative Real-Time Polymerase Chain Reaction Test and Virus Quantification

**Figure 4:**
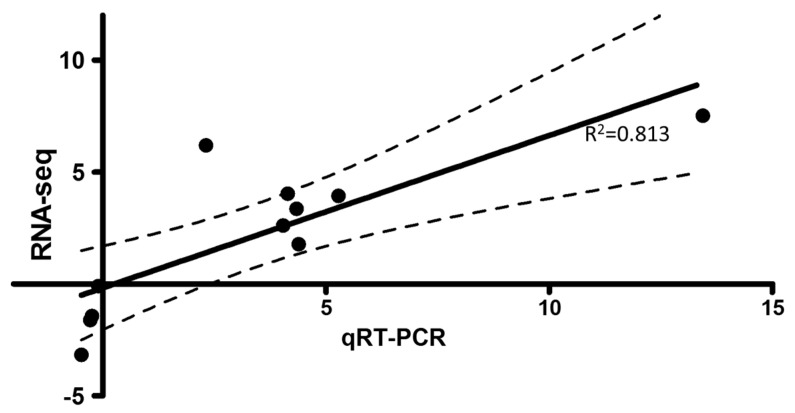
Shows the Pearson’s correlation coefficency (*r*^2^ = 0.813, *p* = 0.0023) of RNA-seq *versus* qRT-PCR. Dots show number of RNA-Seq *versus* qRT-PCR pairs analyzed while the dotted lines show the 95% Confidence Interval of the correlation coefficiency line.

Figure 4 shows randomly selected genes for qRT-PCR analysis of the TLR and RLR pathway genes detected by RNA-seq. In the situation where duplicated copies of the selected genes existed [13,38], only genes that had the highest significant (*p*-value) of expression were used for qRT-PCR analysis. Among the TLR genes, TLRs 3 and 8 were upregulated, while, among the RLR genes, RIG-I, MDA5, LPG2 and IPS-1 were also upregulated. In addition, IRF3, IRF7 and IP-10 reported from both TLR and RLR pathways were also upregulated. Genes that were downregulated included the toll-interacting protein (TOLLIP), TRAF6, p38b1 mitogen activated protein kinase (p38) and ras-related protein rac1 (Rac1). Overall, Figure 4 shows genes that genes that were up- or downregulated by RNA-seq were also up- or downregulated by qRT-PCR thereby confirming the validity of our RNA-seq data. In terms of virus quantification, SAV-3 was only detected in the infected cells at mean Cp value 20 (*n* = 3) of the E2 structural protein detected by qRT-PCR. E2 gene transcription was not detected by qRT-PCR in the non-infected cells.

## 4. Discussion

### 4.1. Transcriptome Signaling Pathway Analysis

Gene ontology (GO) and KEGG are among the most commonly used databases for functional annotation. While GO terms are mostly used for the annotation of individual genes [39], KEGG pathways are widely used for annotations in which genes can be grouped into network maps [39,40], which provides a functional understanding of genes that work together in a pathway. KEGG pathway maps can be plotted into biological pathways of model and none-model organisms [40,41]. Based on statistical analyses, pathways with significant enrichment can be determined to ensure that only pathways having relevant biological implications are used in the analysis of transcriptome data. Thus, pathways identified as the most enriched can be useful in identifying relevant genes activated in response to stimuli while genes that rank high in a pathway could serve as potential candidates for testing the validity of the pathway using functional studies such as the use of knock-out-models. Hence, in the case of duplicated genes, isoforms having the highest significance (*p* = value) of differential expression were used for the validation of RNA-seq data by qRT-PCR because they were considered to have the highest impact on influencing the outcome of the PRR network pathways induced by SAV-3 infection in TO-cells. In this study, the KEGG pathway analysis showed significant enrichment of the endosomal TLRs and RLRs and not the NLRs in TO-cells infected by SAV3. As such, further analysis paved the way to deciphering the pathway network of genes involved in TLR and RLR signaling expressed in response to SAV-3 infection in TO-cells as shown below. Detection of increased levels of the E2 structural protein in TO-cells 48 h post infection by qRT-PCR consolidates the fact that the TLR and RLR genes differentially expressed in this study were induced by SAV-3 infection in TO-cells. Hence, the pathway analysis carried out in this study suggests that SAV-3 infection could be using the TLR and RLR pathways to produce high levels of type I IFNs in TO-cells. Overall this study shows that pathway based analyses improves the analytical power to identify the most important genes expressed in response to stimuli in a *de novo* assembled transcriptome. Further, the study also shows that the use of pathway based analysis to decipher molecular networks of genes expressed in a transcriptome provides a contextual understanding of biological processes induced by microbial invasion unlike the tedious work of trying to identify the biological functions of individual genes expressed in a transcriptome using a non-systematic approach.

### 4.2. Toll-Like Receptor Signaling Genes

The TLR family is made of extracellular and intracellular receptors able to recognize PAMPs from different pathogens [42,43]. In the present study, only TLRs 3 and 8 were upregulated suggesting that recognition of SAV-3 in TO-cells could be by the endosomal TLRs and not the cell surface TLRs. Studies in mammals have shown that TLR8 is only expressed by phagocytic cells such as macrophages and dendritic cells (DCs) [44] while TLR3 has been shown to be a dsRNA innate immune receptor primarily expressed by macrophages and DCs [28]. Put together these observations support observations made by Pettersen *et al.* [19] who showed that TO-cells derived from Atlantic salmon leukocytes possess macrophage/dendritic cell like properties and, hence, their ability to express both TLRs 3 and 8 in response to SAV-3 infection further firms up this notion. In fish, TLR3 is expressed in high levels in different organs inclusive of mucosal and lymphoid organs suggesting that it could play an important role in sensing pathogens at portals of entry in mucosal organs as well as recognizing pathogens that reach the lymphoid organs after entering the systemic environment [45,46,47]. On the contrary, TLR8 is mainly expressed in lymphoid organs [17,38,48] suggesting that it plays a vital role in sensing viruses that get to lymphoid organs via APCs. Studies in humans have shown that TLR8 binds to viral ssRNA [26] suggesting that it could be using similar mechanisms to bind to the ssRNA genome of SAV-3 in TO-cells. On the other hand, TLR3 has been shown to be specific for the recognition of viral dsRNA [28]. This is supported by observations made by Weber et at [49] who showed that viral ssRNA produce intermediate dsRNA which is recognized by TLR3 and given that alphaviruses replicases form dsRNA intermediates as pointed by Smerdou *et al.* [50], it is likely that the sensing of SAV-3 infection by TLR3 in TO-cells could be by recognition of the intermediate dsRNA produced in its replicative form. However, there is need for detailed investigations to support these observations given that such information has proved to be useful in the targeting of alphavirus replicons in APCs for the optimization of vaccine performance as shown in the case of mammalian alphaviruses [51,52,53]. In terms of downstream signaling, upregulation of TLR3 and TLR8 was linked to upregulation of IRF3 and IRF7 culminating in upregulation of IFN-a2 which is in line with observations seen in higher vertebrates that TLR3 and TLR8 produce type I IFNs via IRF3 and IRF7 [54,55]. In addition, the study also shows that activation of IFN-α/β receptors was linked to upregulation of STAT1 via the JAK/STAT pathway resulting in upregulation of IP-10 and I-TAC, which are chemoattractants for T-cell responses in virus infected cells [56]. All in all, the repertoire of *TLR* genes expressed by TO-cells in response to SAV-3 infection conforms to genes expressed by the endosomal TLRs signaling pathways expressed in mammalian cells suggesting that fish macrophages and DC-like cells could be using similar mechanisms to those used by mammalian macrophages and DCs to combat intracellular microbial invasion [9,54,57,58]. However, there is need for detailed studies using knockout systems to demonstrate the functional mechanisms of individual genes expressed in the TLR signaling pathway shown in this study.

### 4.3. RIG-I-Like Receptor Signaling Genes

Although the significance of antiviral effects of TLRs 3 and 8 in macrophages and DCs is indisputable, the key viral sensors for other cell types for intracellular recognition of infection are RLRs [59]. In the case of ssRNA viruses, the major PAMP recognized by RLRs is the 5′-triphosphate (ppp-) RNA [60,61,62]. Single stranded 5′-ppp-RNAs that lack 2’-O-methylation of the 5′-cap, but bear a 5′-ppp-RNA, are specifically from viruses, which serve as a molecular signature for distinguishing self from non-self mRNAs [63,64]. This PAMP is recognized by RIG-I and it is expressed by several viruses including alphaviruses [56] suggesting that the sensing of SAV-3 by RIG-I in this study could be through the same PAMP used to bind to RIG-I in the cytosol for mammalian viruses [60,61,62]. Although RIG-I signaling is dependent on 5′-ppp-RNA binding, it requires ubiquitination by TRIM25 [65], oligomerization by MITA [60] and IPS-I multimerization on the mitochondrion-associated endoplasmic reticulum [66,67], which could account for the expression of these genes in this study. Apart from the 5-ppp-RNA PAMP, the presence of viral dsRNA in host cells is recognized as a non-self-entity given that vertebrate cells do not encode the RNA-dependent-RNA-polymerase (RdRp) encoded in RNA viral genomes [27]. Unlike RIG-I that detects the 5′-ppp-RNA PAMP, MDA5 functions as a sensor for dsRNA in the cytosol [29,30,68]. As pointed out by Nikonov *et al.* [27], the +ssRNA of alphaviruses serves as an mRNA that is transcribed to form a dsRNA in the cytosol during replication. Given that both RIG-I and MDA5 bind to dsRNA, it is likely that the dsRNA formed during virus replication serves as a ligand that binds to RIG-I and MDA5. Hence, upregulation of MDA5 in this study could be linked to detection of dsRNA generated from SAV-3 replication in TO-cells, which conforms to observations made for other alphaviruses [27]. Expression of MDA5 and RIG-I has been shown to increase following infection by dsRNA viruses such as infectious pancreatic necrosis virus (IPNV) in Atlantic salmon [69] suggesting that these PRRs could play an important role in virus recognition by APCs and lead to their activation to enhance antigen uptake, processing and presention for activation of the adaptive immune system [70]. On the other hand, LGP2 has been shown to potentiate the function of RIG-I while blocking the function of MDA5 in mammals [71,72]. The KEGG pathway analysis used in this study shows that the RIG-I and MDA5 pathways converge on the IPS-I adaptor, which is in line with observations seen in mammals [73,74]. Similar to observations made for TLR3 and TLR8 signaling pathways above, the RLR signaling pathway analysis carried out in this study shows that downstream signaling via IPS-1 culminate in production of type I IFNs using the IRF3 and IRF7 signaling pathways which is in line with observations seen in higher vertebrates [27,52,73]. Overall, the repertoire of genes clustered in the RLR signaling pathway generated in this study conforms to genes induced by other alphaviruses in higher vertebrate dendritic cells [75] suggesting that SAV-3 infection in TO-cells uses similar mechanisms to produce type I IFNs and anti-inflammatory cytokines used to combat alphavirus infection in higher vertebrates.

## 5. Conclusions

In this study, we have shown that the repertoire of genes linked to PRRs induced by SAV-3 infection in TO-cells is comparable to genes induced by other alphaviruses in Mammalia [66]. Among the TLRs, only endosomal TLRs 3 and 8 were upregulated while RIG-I, MDA5 and LPG2 were upregulated among the RLRs suggesting that these PRRs are essential for the sensing of SAV-3 infection in TO-cells. Both TLR and RLR signaling pathways were linked to upregulation of IRF3 and IRF7 culminating in upregulation of IFN-a2. This study links the expression TRIM25 to the RIG-I signaling pathway being the first report that points to the possible involvement of this gene in the recognition of SAV-3 infection in TO-cells. Finally, upregulation of IFN-a2 observed from the TLR and RLR pathways suggest that SAV-3 functions through these pathways for potent induction of IFN-a2. It is important to point out that data generated here is based on transcriptome analysis of different genes expressed in response to SAV-3 infection in TO-cells, there is need for further studies to consolidate these findings using functional studies such as gene knockout systems to elucidate the functional activities of all the genes expressed in this study. Overall, the study shows that a pathway-based approach improves the analytical power of transcriptome data analysis and that it provides a contextual approach to understanding the biological relevance of DEGs induced by microbial invasion.

## Figures and Tables

**Table 1:** Primers used for quantitative real time PCR.

Primer Name	Sequence	GeneBank Accession No.
SAV-3 E2-F	CAGTGAAATTCGATAAGAAGTGCAA	EF675594
SAV-3 E2-R	TGGGAGTCGCTGGTAAAGGT	
β-Actin-F	CCAGTCCTGCTCACTGAGGC	AF012125
β-Actin-R	GGTCTCAAACATGATCTGGGTCA	
IP-10-F	TGCCAGAACATGGAGATCAT	EF619047
IP-10-R	TTTACTGCACACTCCTTTGGTT	
TLR3-F	TTTGATGAGTCTCCGCCAACTCCA	KP231342
TLR3-R	AATCTGCGAGGGACACAAAGGTCT	
TLR8-F	ACAAGAAAGAATGCCTCAATGTCA	NM_001161693
TLR8-R	CACCCAGTCTGACACCAACA	
IRF3-F	TGGACCAATCAGGAGCGAAC	FJ517643
IRF3-R	AGCCCACGCCTTGAAAATAA	
IRF7-F	GAGGAGTGGGCAGAGAACTA	NM_001171850
IRF7-R	TTCTGGGAGACTGGCTGGG	
STAT1-F	CGGGCCCTGTCACTGTTC	GQ325309
STAT1-R	GGCATACAGGGCTGTCTCT	
RIG-I-F	GACGGTCAGCAGGGTGTACT	NM_001163699
RIG-I-R	CCCGTGTCCTAACGAACAGT	
MDA5-F	AGAGCCCGTCCAAAGTGAAGT	NM_001195179
MDA5-R	GTTCAGCATAGTCAAAGGCAGGTA	
LGP2-F	GTGGCAGGCAATGGGGAATG	FN396358
LGP2-R	CCTCCAGTGTAATAGCGTATCAATCC	
TOLLIP-F	ACCATTAGCACCCAACGAG	BT045489
TOLLIP-R	TGGGAGTAATACGCAGGAAG	
RAC1-F	GACAGGAAGACTACGACAGAC	NM_001160673
RAC1-R	TCAAAGGAGGCAGGACTCAC	
TRAF6-F	ACAGACTGTCCAAAGGCTC	–
TRAF6-R	TCATTGCGCTGCATCATC	
P38-F	TCCACGCCAAGAGAACCTAC	NM_001123715
P38-R	ACATCATTGAACTCCTCCAGAC	

**Table 2:** KEGG analysis of the pattern recognition receptors induced by SAV-3 infection in TO-cells.

Parameters	Toll Like Receptor	RIG-I-Like Receptor	NOD Like Receptor
Pathway ID	Ko04620	Ko04622	ko04621
Pathway significance	0.058	0.024	0.9101
Pathway enrichment	4.865157 × 10^−1^	3.011733 × 10^−1^	1.00000 × 10^0^
Total KO genes	20115	20115	20115
All genes with pathway annotation	9315	9315	9315
All genes in each pathway	216	144	212
DEGs	112	79	89

**Table 3A:** Toll like receptor pathway genes upregulated in response to SAV-3 infection in TO-cells.

Gene Name	Abbr.	NCBI	Unig	KO	Reg	Log_2_ ratio	*p*-Value
*Toll like receptor 3*	TLR3	|DAA64469.1|	Unig9113	K05401	Up	2.6140	7.4290 × 10^−71^
*Toll like receptor 8*	TLR8	|NP_001155165.1|	Unig2363	K10170	Up	4,0462	3.4201 × 10^−5^
*Signal transducer and activator of transcription 1*	STAT1	|NP_001134757.1|	CL2066.2	K11220	Up	6.27213	3.0554 × 10^−68^
*Interferon regulatory factor 3*	IRF3	|ACL68544.1|	Unig4271	K05411	Up	3.3644	5.3137 × 10^−135^
*Interferon regulatory factor 7*	IRF7	|NP_001165321.1|	Unig10251	K09447	Up	3.1970	1,13523 × 10^−22^
*Interferon α*	IFN-a2	|NP_001117042.1|	Unig5589	K05414	Up	7.6042	–
*Interferon α receptor 1*	IFNAR1	|NP_001268239.1|	Unig34816	K05130	Up	1.8640	8.9299 × 10^−66^
*IFNγ induced protein 10*	IP-10	|ACI69209.1|	Unig8163	K12671	Up	7.5233	2.2267 × 10^−112^
*IFN-inducible T-cell* *α* *chemoattractant*	I-TAC	|NM_0011412293.1|	Unig1740	K12762	Up	9,55672	–

**Table 3B:** Toll like receptor pathway genes downregulated during SAV-3 infection in TO-cells.

Gene Name	Abbr.	NCBI	Unigene	KO	Reg	Log_2_ ratio	*p*-Value
Receptor interacting serine/threonine protein kinase 1	RIP1	|NP_001036815.1|	Unig17924	K02861	Down	−1.3056	2.0987 × 10^−8^
Caspase 8	CASP8	|XP_001335163|	CL4461.1	K04398	Down	−1.1800	2.4337 × 10^−142^
Toll like receptor 1	TLR1	|ACV92064.1|	Unig41380	K05398	Down	−4.2514	3.3298 × 10^−5^
Toll like receptor 2	TLR2	|CCK73195.1|	Unig9045	K10159	Down	−16589	1.7562 × 10^−41^
Transcription factor AP-1	AP-1	|XP_004369047.1|	CL3191.1	K04448	Down	−2.2025	3.9706 × 10^−9^
Extracellular signal-regulated kinase	ERK	|BAD23843.1|	Unig24550	K04371	Down	−1.8016	1.5449 × 10^−5^
NF-kappa-B inhibitor α	NFκBα	|ACI67986.1|	CL8473.1	K04735	Down	−1.3923	1.6765 × 10^−13^
TANK-binding kinase 1	TBK1	|JF241943.1|	Unig5544	K05410	Down	−1.2619	1.0212 × 10^−124^
TNF receptor associated factor 6	TRAF6	–	Unig40008	K03175	Down	−3.1583	3.552 × 10^−4^
Interleukin 8	IL-8	|NP_001134182.1|	Unig7278	K10030	Down	−1.7368	6.5401 × 10^−93^
Kinase 1-binding protein 1	TAB1	|XP_002662286.2|	Unig1972	K04403	Down	−1.5614	7.0199 × 10^−66^
Kinase 1-binding protein 2	TAB2	|XP_003971436.1|	CL4395	K04404	Down	−2.07925	9.2399 × 10^−28^
Phosphatidylinositol-4,5-bisphosphate 3-kinase	PI3K	|XP_003455769.1|	CL120	K02649	Down	−1,9295	5,7289 × 10^−39^
RAC-α serine/threonine-protein kinase (AkT)	AkT	|ACH70834.1|	CL5806	K04456	Down	−1.43478	2.9777 × 10^−19^
Mitogen-activated protein kinase kinase 6	MKK6	|AAV52830|	Unig80	K04433	Down	−1.6590	7.5296 × 10^−169^
Mitogen-activated protein kinase kinase 4	MKK4	|ACI33552.1|	CL292.2	KO4430	Down	−1.80397	1.9570 × 10^−19^
p38b1 mitogen activated protein kinase	p38	|EF123660.1|	Unig10574	K04441	Down	−1.6142	7.29346 × 10^−9^

**Table 4A:** RIG-I-like receptor pathway genes upregulated in response to SAV-3 infection in TO-cells.

Gene Name	Abbr.	NCBI	Unigene	KO	Reg	Log2ratio	*p*-Value
Retinoic acid-inducible gene-I	RIG-I	|NP_001157171.1|	Unig7848	K12646	Up	3.9377	3.171 × 10^−102^
Melanoma differentiation associated gene 5	MDA5	|NP_001182108.1|	Unig6816	K12647	Up	1.7788	1.8065 × 10^−172^
Laboratory of genetics and physiology 2	LPG2	|NP_001133649.1|	CL8555	K12649	Up	5.5128	0
Interferon promoter stimulating protein 1	IPS-1	|NP_001161824.1|	Uni12389	K12648	Up	1.5588	3.0551 × 10^−18^
Tripartite motif-containing protein 25	TRIM25	|ACN11060.1|	CL8518.2	K10652	UP	4.2080	1.8646 × 10^−5^
IFNγ induced protein 10	IP-10	|ACI69209.1|	Unig8163	K12671	Up	7.5233	2.2267 × 10^−112^
Optineurin	Optn	|NP_001133761.1|	CL4866	K07210	Up	1.9853	0
Interferon regulatory factor 3	IRF3	|ACL68544.1|	Unig4271	K05411	Up	3.3644	5.3137 × 10^−135^
Interferon regulatory factor 7	IRF7	|NP_001165321.1|	Unig8533	K09447	Up	11.055	1.1354 × 10^−22^
Interferon a2	IFN-a2	|NP_001117042.1|	Unig5589	K05414	Up	7.6042	0

**Table 4B:** RIG-I-like receptor pathway genes downregulated during SAV-3 infection in TO-cells.

Gene Name	Abbr.	NCBI	Unigene	KO	Reg	Log2ratio	*p*-Value
NLR family member X1	NLRX1	|AFY26970.1|	Unig11078	K12653	Down	−1.6500	1.5422 × 10^−58^
Autophagy protein 5	Atg5	|ACN11274.1|	Unig5014	K08339	Down	−1.3748	7.961 × 10^−10^
Interleukin 8	IL-8	|ABA86669.1|	Unig7278	K10030	Down	−1.7368	6.5401 × 10^−93^
Ubiquitin carboxyl-terminal hydrolase CYLD	CyLD	|XP_004068277.1|	Unig18460	K08601	Down	−1.9826	4.4252 × 10^−11^
Suppressor of IKK-epsilon	SIKE	|ACI33887.1|	Unig21218	K12656	Down	−1.5344	2.5131 × 10^−7^
TNF receptor type 1-associated death domain	TRADD	|Q1M161.1|	CL8304	K03171	Down	−1.6665	1.8945 × 10^−20^
TGF-β-activated kinase 1	TAK1	|AAT07829.1|	CL7020.1	K04427	Down	−1.1643	3.2707 × 10^−14^
Nuclear factor κ-B	NFκβ	|HM771267|	CL8473.1	K04735	Down	−1.3923	1.6765 × 10^−13^
TANK-binding kinase 1	TBK1	|JF241943.1|	Unig5544	K05410	Down	−1.2619	1.0212 × 10^−124^
TNF receptor-associated factor 2	TRAF2	|NP_001167255.1|	Unig16762	K03173	Down	−1.1445	1.0105 × 10^−4^
NF-κ-B inhibitor	IκB	|CAC85086.1|	Unig31637	K02581	Down	−2.8369	1.9042 × 10^−4^
Caspase 8	CASP8	|XP_001335163|	CL4461.1	K04398	Down	−1.1800	2.4337 × 10^−142^
Caspase 10	CASP10	|CAE51933.1|	CL7349	K04400	Down	−1.2252	2.8250 × 10^−23^
Receptor-interacting threonine-protein kinase 1	RIPK1	|NP_001036815.1|	Unig17924	K02861	Down	−1.3056	2.0987 × 10^−8^
